# Exercise, myonectin response, and insulin resistance among overweight, obese and healthy individuals: a systematic review and narrative synthesis

**DOI:** 10.25122/jml-2024-0277

**Published:** 2025-02

**Authors:** Aysha Mohamed Mohamed Alsahi Alzaabi, Ramprasad Muthukrishnan, Marwan Ismail, Praveen Kumar Kandakurthi, Satheeskumar Durairaj, Shanmugam Sukumar, Kumaraguruparan Gopal

**Affiliations:** 1 Physiotherapy Department, Kalba Hospital, Sharjah, United Arab Emirates; 2 Physiotherapy Division, College of Health Sciences, Gulf Medical University, Ajman, United Arab Emirates; 3 Laboratory Sciences, College of Health Sciences, Gulf Medical University, Ajman, United Arab Emirates

**Keywords:** prediabetes, obesity, overweight, myokine, exercise

## Abstract

Myonectin, a novel muscle-derived peptide or myokine, has been implicated in glucose and lipid homeostasis through its autocrine, paracrine, and endocrine functions. This review aimed to explore the impact of structured exercise interventions on myonectin levels and insulin resistance indicators in healthy individuals and those living with overweight or obesity. We performed a search of PubMed, Science Direct, CINAHL, TRIP Database, Cochrane Library, and Google Scholar for studies published until July 2022. The key terms used were “prediabetes”, “overweight”, “obesity”, “myonectin”, “Complement 1q / tumor necrosis factor-related protein 5 (CTRP5 or C1qTNF5)”, “erythroferrone”, and “exercise”. Eight studies investigated the effects of exercise on myonectin levels and insulin resistance, measured through the Homeostatic Model Assessment for Insulin Resistance (HOMA-IR), in individuals who were overweight or obese, while six studies focused on those without these conditions. After data extraction, narrative qualitative synthesis and risk of bias analysis were performed. Findings indicate that structured aerobic or combined aerobic and resistance training at moderate intensity over 8–12 weeks led to significant increases in myonectin levels and reductions in insulin resistance, particularly among women who were overweight or obese. However, data was limited by heterogeneous age and gender groups' metabolic profiles and variability in exercise protocols. Myonectin response to exercise in healthy adults remains unclear due to baseline metabolic variability, though some improvements in the glucose-insulin axis were noted. This review suggests that myonectin may serve as a valuable biomarker to assess the impact of exercise on insulin sensitivity in individuals at risk of diabetes with overweight or obesity.

## INTRODUCTION

Aerobic exercise is a cornerstone in the prevention and management of type 2 diabetes mellitus (T2DM), often prescribed alongside other interventions to improve glycemic control and cardiovascular health [1]. Regular physical activity has been shown to reduce glycated hemoglobin (HbA1c), a critical marker of glycemic control, with studies reporting up to a threefold reduction in HbA1c levels among individuals aged 60–80 years after resistance training compared to non-exercising controls [2-4].

During aerobic exercises, skeletal muscles contract and release numerous cytokines or peptides weighing ~5–20 kDa or proteoglycan peptides called myokines [5]. Two of the largest endocrine organs in the body are the skeletal muscle and adipose tissue, which secrete myokines and adipokines, respectively [6]. Recent studies suggested that exercise-induced myokines could offer protection against many chronic diseases, particularly T2DM [7]. Myokines improve muscle metabolism and function through autocrine, paracrine, and endocrine mechanisms, affecting various organs, including the liver, pancreatic β-cells, adipose tissue, and the nervous system [6−9]. However, in individuals with T2DM, myokine secretion patterns may differ. For example, interleukin 6 (IL-6), as a pro-inflammatory cytokine, negatively regulates the acute phase response and is responsible for low-grade chronic inflammation [10,11]. Moreover, the myokine profile of primary human skeletal muscle cells (hSkMCs) in patients with T2DM is different from that of insulin-sensitive hSkMCs [12].

Myonectin, also reported as C1q/TNF-related protein 5 (CTRP-5), C1q/TNF-related protein-15 (CTRP-15), C1q/TNF-related protein isoforms 5, erythroferrone (ERFE) was detected as a novel myokine, and is primarily expressed in skeletal muscles in response to exercise [13,14]. Myonectin is a nutrient-sensing myokine, a carbohydrate or lipid regulator, and is also secreted by skeletal muscle in response to changes in the cellular energy state resulting from glucose or fatty acid fluxes [13]. Myonectin was hypothesized to enhance protein synthesis and inhibit protein degradation by sensing cellular energy state and mitochondrial biogenesis [15]. Depending on the muscle fiber types, myonectin expression may be regulated differently [13,15]. A largely slow-twitch, oxidative fiber type (Type I), such as soleus, tends to have a higher level of myonectin transcript expression relative to a glycolytic, fast-twitch fiber type (Type II) such as plantaris muscle [15].

Some studies suggest that elevated myonectin levels in newly diagnosed T2DM cases may represent a compensatory mechanism to counteract insulin resistance [16]. Myonectin concentrations have been positively associated with measures of insulin resistance and negatively associated with insulin sensitivity, highlighting its role as a metabolic regulator linked to sarcopenic obesity [14,17,18]. Elevated myonectin levels in individuals newly diagnosed with T2DM remained unchanged following 45-minute exercise, lipid infusion, oral glucose challenges, or short-duration physical activity in young, healthy populations [16,17]. This review revolves around many research questions, as mentioned below.

Myonectin can be secreted outside of exercise, possibly in response to other metabolic stresses, which raises questions about its specificity as an exercise-related marker. For example, obesity can increase myonectin levels, which might conflict with its use as a straightforward indicator of healthy exercise response. Can changes in myonectin levels be reliably used as a marker of exercise-induced improvements in metabolic health and diabetes risk? Furthermore, the effects of exercise on myonectin levels across individuals with varying metabolic statuses and responses to physical activity remain poorly understood. While some studies have shown that myonectin levels can increase with exercise, there is variability in the evidence regarding the consistency of this response. The extent to which myonectin levels rise with exercise may differ among individuals, potentially limiting its usefulness as a reliable marker across diverse populations, making it a topic of ongoing debate. In order to shed light on myonectin levels and diabetes risk reduction, it is essential to establish whether individuals without diabetes exhibit any relationship between myonectin levels and insulin resistance. This study explored the uncertainty surrounding the potential link between myonectin and insulin resistance in non-diabetic individuals, investigating its role as a possible marker for metabolic health and diabetes prevention.

Elevated myonectin levels may act as a cardioprotective factor mediated by endurance exercise, as reported in studies using mouse models. In these studies, long-term endurance exercise protocols—60-minute sessions performed 5 days per week for 4 weeks—increased circulating and muscle myonectin levels, which helped mitigate the risk of acute myocardial ischemic injury [19]. In contrast, circulating myonectin levels were reduced in individuals with T2DM. Independent predictors of myonectin levels in this population included body mass index (BMI), low-density lipoprotein cholesterol (LDL-C), triglycerides (TG), the Homeostatic Model Assessment for Insulin Resistance (HOMA-IR), and insulin resistance. Myonectin levels decreased due to elevated levels of circulating free fatty acids (FFAs) and ectopic accumulation into other tissues, which might lead to clinical complications [13]. A decrease in myonectin levels was also reported in nephropathy [20] and peripheral artery disease [21] in individuals with T2DM.

A conflict arises regarding how muscle types, such as slow-twitch versus fast-twitch fibers, and overall muscle mass influence myonectin production. Larger, more active muscles may release greater amounts of myonectin, but this may not apply uniformly across individuals, including those with varying levels of obesity and overweight. Whether this means that certain people are inherently better able to lower their diabetes risk through exercise due to their muscle composition and their exercise effects on myokines, particularly myonectin, remains a point of debate.

There are no conclusive findings on long-term exercise-induced changes in myonectin levels in humans. Limited studies have explored the relationship between exercise-induced myonectin levels and glycemic control or insulin resistance, particularly in individuals with overweight or obesity. The exercise duration needed to elicit changes in myonectin levels and its effects on metabolic risk factors remains poorly understood. Hence, studying uncertainties surrounding a) myonectin levels pre and post-exercise intervention in individuals with varying metabolic statuses, b) the impact of exercise duration, c) type of exercise for targeting muscles, and finally, d) myonectin-mediated effects on glucose and insulin resistance in healthy individuals or those at risk for early detection of diabetes, are crucial to deepen our understanding of metabolic linked pathogenesis and identify potential therapeutic exercise targets.

A systematic literature review approach was chosen to explore missing clues and identify exercise prescription factors that could mitigate or clarify the risk of transitioning from overweight or obesity to the prediabetes stage, focusing on the role of the myonectin axis.

The research question was:

How do exercise-induced changes in myonectin levels relate to improvements in insulin resistance in healthy, overweight, and obese individuals? This systematic review aimed to investigate whether exercise-induced changes in myonectin levels were associated with improved insulin resistance in healthy, overweight, and obese individuals and identify exercise-specific myonectin responses linked to myonectin-mediated glucose metabolism.

## MATERIAL AND METHODS

### Search strategy

An online search was conducted using the following databases: PubMed, Science Direct via Scopus, Cumulative Index of Nursing and Allied Health Literature (CINAHL) via EBSCO, TRIP Database (Turning Research into Practice), Cochrane Library, and Google Scholar. The search focused on medical terms such as “prediabetes”, “overweight”, and “obesity”, combined with key terms including “myonectin”, “Complement C1q Tumor Necrosis Factor-Related Protein or isoform 5: ‘CTRP5’ or ‘C1QTNF5’”, “Erythroferrone”, and “exercise” using Boolean search operators. The main keywords, full search strategy, and syntaxes are provided in a supplementary file. The database search was limited to studies published between 2010 and July 2022, including human participants. Registration: PROSPERO: CRD42020138434.

Supplementary File

### Selection criteria

Studies that evaluated the effects of exercise interventions on myonectin responses and insulin resistance in overweight and obese individuals were selected. In addition, studies reporting CTRP5/C1QTNF5 protein family expression and secretion from myocytes or exercise intervention with glucose or lipid metabolism were selected. Two authors, MRP and AMAA, independently searched and scrutinized the articles. The PICO (Population, Intervention, Comparison, and Outcome) framework was used as follows:

- **Population:** studies involving healthy adults and overweight and obese individuals aged 18 years and older were included. Studies involving individuals with systemic diseases or injuries were excluded.
- **Intervention:** the review included aerobic and/or resistance exercise interventions lasting at least 8 weeks for overweight and obese participants, with comparisons to healthy adults participating in aerobic and/or resistance exercise without a defined duration limit. Interventions such as Pilates, body-mind movement, stretching, breathing exercises, and meditation were excluded.
- **Comparison:** studies were required to include pre-exercise and post-exercise measurements. Studies lacking baseline or follow-up data for overweight and obese participants were excluded.
- **Outcome:** studies assessing changes in blood serum or plasma myonectin levels and insulin resistance parameters in overweight and obese participants were included.

The review focused on comparative exercise intervention studies, including randomized and non-randomized clinical trials published in English or Arabic in peer-reviewed journals. Exclusion criteria included animal studies, very short-term exercise interventions (post-analysis conducted within 24–48 hours), diagnostic studies, case reports, review articles, letters, conference abstracts, theses, book chapters, and guidelines.

### Data extraction and study appraisal

Articles meeting the inclusion criteria checklist were reviewed by two authors who reached a consensus and independently extracted data. Two authors (SK & KG) re-checked and confirmed the data obtained. The extracted data included details on overweight and obese participants, exercise interventions, and myonectin levels, followed by glucose and insulin parameters, stored in Word documents and Excel files as SI units.

The following exercise parameters were derived and tabulated for each study: a) type of exercise, b) duration, c) number of sessions per week, d) total intervention period (in weeks), e) time spent, and f) intensity of exercise.

The anthropometric and biochemical parameters measured before and after exercise training were thematically categorized into groups based on participant characteristics (overweight and obese individuals or healthy participants) and the duration of exercise intervention (more than 10 weeks and less than 8 weeks).

The Risk of Bias in Non-Randomized Studies of Interventions (ROBINS-I) tool was used to ensure the quality and reliability of non-randomized studies by systematically identifying and addressing various sources of bias. Each element of the tool was assessed independently by two reviewers, and a consensus was reached to derive a cumulative rating [22].

A narrative synthesis and review were developed based on two themes: a) myonectin response levels before and after exercise and b) the effects or associations of myonectin with glucose tolerance and insulin resistance in overweight, obese, and healthy participants. The results of individual studies were summarized to draw overall inferences. A consensus among the authors was reached to provide a main summary of the results based on the following factors: a) exercise interventions lasting up to 12 weeks, b) up to 8 weeks, and the type of exercise intervention.

### Reporting

The present literature review adheres to the Preferred Reporting Items for Systematic Reviews and Meta-Analyses (PRISMA) guidelines to ensure transparency and completeness in reporting the review process and findings [23].

## RESULTS

### Article selection process

**Figure 1:**
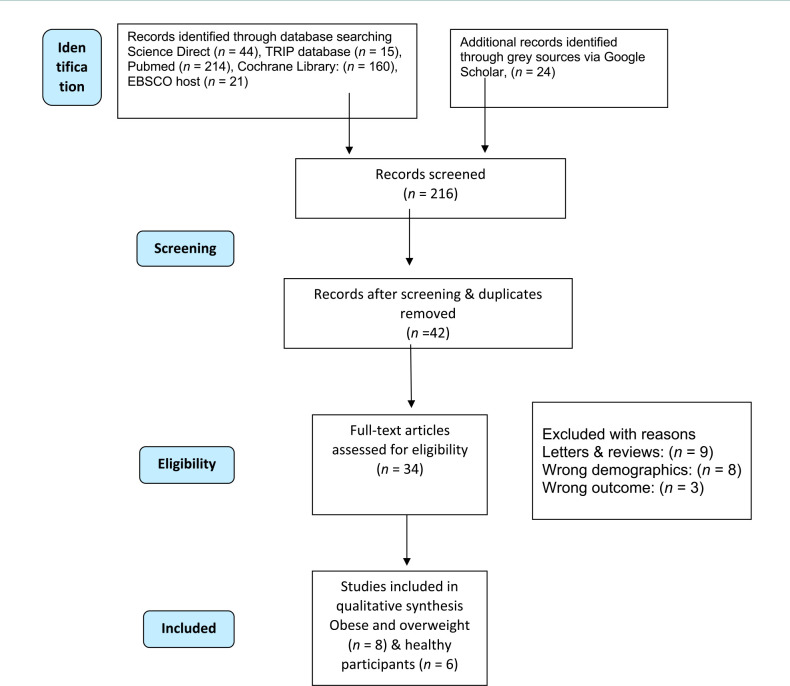
Article selection flow diagram

A total of 1,231 articles were initially retrieved from electronic databases and subjected to manual screening for relevance. Reviewers carefully assessed each article to determine whether they met the predefined inclusion and exclusion criteria, resolving any disagreements through consensus. Following this process, duplicate articles were identified and removed, leaving 478 unique articles for screening. Subsequent screening and further evaluation based on the PICO checklist and removing duplicates resulted in 42 articles. Full eligibility criteria were met by the 14 full-text articles included for a comprehensive review. The selection process adhered to PRISMA statement guidelines, ensuring transparency and reproducibility. The flowchart illustrating the article selection procedure is presented in Figure 1 [23].

A checklist of inclusion criteria, exercise dosage parameters, demographic details with changes in bio-metabolic variables, and a summary of included articles of obese and overweight participants are presented in Tables 1–4. Tables 3 and 4 summarize the effects of exercise on myonectin up to 8 weeks and 12 weeks, respectively [24-31]. Similar details are extracted in Tables 5–7 for healthy participants [32-37].

**Table 1:** Summary of included studies using the PICO framework for exercise interventions in overweight and obese individuals (*n* = 8)

Author, Year	Study Design	Overweight	Obese	Age > 18 years	BMI ≥ 25 Kg/m^2^	Control group	Exercises groups	Myonectin level	Adiponectin	FPG	FPI	Insulin resistance*	Total cholesterol
Lim *et al.*, 2012	Quasi-experimental and cross-sectional	✓	-	✓	-	-	✓	✓	✓	✓	✓	✓	✓
Choi *et al.*, 2013	Quasi-experimental	-	✓	✓	✓	-	✓	✓	✓	✓	✓	✓	✓
Pourranjbar *et al.*, 2018	Quasi-experimental and cross-sectional	-	✓	✓	✓	✓	✓	✓	-	✓	✓	✓	-
Kazemi *et al.*, 2015	NA	✓	✓	✓	✓	✓	✓	✓	-	✓	✓	-	-
Safarzade *et al.*, 2017	NA	-	✓	✓	✓	✓	✓	✓	-	✓	-	✓	✓
Bagheri *et al.*, 2018	Semi-experimental with pre-test and post-test design	✓	✓	✓	✓	✓	✓	✓	-	✓	✓	✓	-
Zefreei *et al.*, 2020	NA	-	✓	✓	✓	✓	✓	✓	-	✓	✓	✓	-
Safarpour *et al.*, 2021	NA	-	✓	✓	✓	✓	✓	✓	-	✓	✓	✓	-

BMI, Body Mass Index; FPG, Fasting Plasma Glucose; FPI, Fasting Plasma Insulin; *HOMA-IR, Homeostatic Model Assessment for Insulin Resistance

**Table 2:** Type of exercise and exercise dosage parameters in overweight and obese individuals

Study	*n*	Experimental group	Control group	Type of exercise	Duration (Week)	Duration (Time)	Number of Sessions /Week)	Intensity
Lim *et al.*, 2012	28	Older overweight women* (*n* = 14)	Healthy younger women (*n* = 14)	Aerobic exercise	10 weeks	1 hour	3x/week	AE at 60-80% up to 95% of maximum oxygen consumption (VO_2_ max), or above 89% of HRmax
Choi *et al.*, 2013	453	Overweight women (*n* = 76)	None	Combined resistance and aerobic exercise	12 weeks	~ 75 minutes	5x/week	Brief warm-up, ~45 minutes AE at 60-75% age-predicted HRmax, followed by 20-minute strength training and a brief cool-down
Pourranjbar *et al.*, 2018	80	Obese women* (*n* = 34)	Obese women* (*n* = 46)	Aerobic exercise	8 weeks	45 minutes	3x/week	AE, running at 50-70% of HRmax
Kazemi *et al.*, 2015	40	Overweight women Endurance-resistance (*n* = 9), resistance-endurance (*n* = 10), interval resistance-endurance training (*n* = 12)	Overweight women (*n* = 9)	Concurrent training (strength & endurance)	8 weeks	60	3x/week	Progressive training: Starting with low to high intensity. Includes exercise bike (6-65% HRmax, ~45-50% VO_2_ max, 16 min in week 1), resistance training progressively increased to 60-85%.
Bagheri *et al.*, 2018	28	Overweight and inactive women (*n* = 14)	Overweight and inactive women (*n* = 14)	HIIT	8 weeks	~30-45	3x/week	Interval running at maximum speed.
Safarzade *et al.*, 2017	23	Obese men (*n* = 12)	Obese men (*n* = 11)	Resistance training	8 weeks	~60	3x/week	Circuit-based training at 50-85% 1RM (12 stations, 8-10 repetitions; load progression from 50% to 85% 1RM weekly)
Zefreei *et al.*, 2020	40	Obese women group (1) Aerobic/E (*n* = 10)	Obese women Group (4) (*n* = 10)	Aerobic exercise	8 weeks	30-45	5x/week	65%-70% of maximum heart rate.
Safarpour *et al.*, 2021	24	Overweight women (*n* = 12)	Overweight women (*n* = 12)	High-intensity interval swimming training	8 weeks	25-30	3x/week	Warm-up, 6-10 repetitions of 30 seconds of high-intensity front crawl swimming and 10 minutes of cool-down.

E, Aerobic Exercise; HRmax, Maximum Heart Rate; NP, Not Provided; RM, Repetition Maximum; TC, Total Cholesterol; VO_2_ max, Maximal Oxygen Uptake

**Table 3:** Anthropometric and biochemical parameters before and after exercise training in obese and overweight individuals with exercise participation duration of more than 8 weeks (10-12 weeks, *n* = 2)

Study description				Body weight (Kg)		BMI (Kg/m^2^)		Myonectin		Adiponectin (µg/ml)		Fasting Glucose		Fasting Insulin (µU/mL)		Insulin resistance			TC		Overall inference after exercise	
Author/year	Duration	Age	Groups	Before	After	Before	After	Before	After	Before	After	Before	After	Before	After	Before	After	Before		After		
Lim *et al.*, 2012	10 weeks	(22.5 ±2.7 yr)	Group 1: Healthy younger women (*n* = 14)	NP	NP	22.2 ± 1.8	21.3 ± 2.5 ↓	58.37 ± 10.93	46.59 ± 13.99*↓	7.9 ±2.3	9.8 ± 4.2*↑	84.9 ± 11.8 (mg/dL)	73.6 ±6.0* (mg/dL) ↓	10.2 ± 2.7	8.8 ± 3.4*↓	1.9 ± 0.4	1.8 ± 0.7*↓	179.2 ± 15.9 (mg/dl)		173.1 ± 20.7* (mg/dl) ↓	Myonectin levels, BMI, fasting glucose, insulin, IR and TC ↓, adiponectin levels ↑.
		(60.3 ± 5.2 yr)	Group 2: Older overweight women (*n* = 14)	NP	NP	25.4 ± 2.9	23.9 ± 2.7*↓	62.75 ± 14.55	46.66 ± 16.99*↓	12.7 ± 5.9	15.7 ± 5.5*↑	87.9 ± 8.2 (mg/dL)	79.3 ± 5.0* (mg/dL)↓	13.0 ± 3.0	9. 9 ± 3.1*↓	2.8 ± 0.9	2.0 ± 0.6*#↓	211.0 ± 24.2 (mg/dl)		190.1 ± 27.9* (mg/dl) ↓	Myonectin levels, BMI, fasting glucose, and fasting insulin ↓, adiponectin levels ↑.
Choi *et al.*, 2013	12 weeks	(30-60 yr)	(*n* = 76)	67.0 (62.0–71.5)	61.8* (57.4–67.2) ↓	26.8 (25.4–29.0)	24.8 *(23.2–26.8) ↓	34.1 ng/ml (28.6–44.3)	38.4 * ng/ml (29.8–55.1) ↑	10.5 (8.0–14.2)	10.8 (7.8–13.7)↑	4.7 (4.2–5.1) (mmol/L)	4.2 *(3.2–4.9) (mmol/L) ↓	NP	NP	1.4 (1.1–1.7)	1.3*(0.8–1.6) ↓	4.5 ± 1.1 (mmol/L)		3.8 ±1.1* (mmol/L) ↓	Myonectin and adiponectin levels ↑ , body weight, BMI, fasting glucose, IR, and TC ↓

BMI, Body Mass Index; Yr, year; FPG, Fasting Plasma Glucose; FPI, Fasting Plasma Insulin; IR, Insulin Resistance; TC, Total Cholesterol; - not reported;

* Significant at P <0.05, IR,# Insulin resistance, value greater than 2 indicates insulin resistance, ^ arbitrary units, arrows indicate increase or decrease.

**Table 4:** Anthropometrics and biochemical parameters before and after exercise training in obese and overweight individuals with exercise participation duration of 8 weeks (*n* = 6)

Study description				Body weight (Kg)		BMI (Kg/m^2^)		Myonectin		Fasting Glucose (mg/dL)		Fasting Insulin (µU/mL)		HOMA-IR		TC	Body fat percentage	Overall inference fter exercising
Author, year/Participants	Duration	Age	Groups	Before	After	Before	After	Before	After	Before	After							
Kazemi *et al.*, 2015 Obese and overweight elderly women	8 weeks	50–70 years old	Endurance-resistance *n* = 9	74.66 ± 4.68	72.77 ± 4.67*	29.89 ± 1.20	29.12 ± 1.20*	504.77 ± 168.27 µg/mL	528.22 ± 100.99 µg/mL	108.33 ± 9.54	93.11 ±5.81	4.38 ± 1.8	3.61 ± 1.58	NP		NP	NP	Exercise groups: Non-significant Myonectin levels ↑, Body weight*, BMI, fasting glucose, and fasting insulin ↓
			Resistance-endurance *n* = 10	70.80 ± 3.90	68.60 ± 3.86*	29.23 ± 1.71	28.30 ± 1.54*	538.00 ± 176.49 µg/mL	586.10 ± 102.84 µg/mL	92.90 ± 1.39	83.50 ± 2.36*	3.77 ± 1.16	3.48 ± 1.3					
			Interval resistance endurance training *n* = 12	66.41 ± 2.69	64.41 ± 2.44*	27.57 ± 0.92	26.76 ± 0.86*	509.66 ± 172.81 µg/mL	518.75 ± 117.25 µg/mL	110.91 ± 8.14	95.41 ± 3.90	4.08 ± 1.76	3.32 ± 1.79					
			control *n* = 9	76.88 ± 3.78	76.66 ± 4.05	31.75 ± 0.91	31.63 ± 1.01	515.55 ± 182 µg/mL	512.77 ± 136.19 µg/mL	117.77 ± 10.60	110.22 ± 9.91	4.46 ± 1.44	4.5 ± 1.19					Control group: Myonectin levels ↓,
Pourranjbar *et al.*, 2018 Obese and overweight women	T1: 8 weeks	(38.15 ± 2.33 yrs)	Aerobic exercise group *n* = 34	NP	NP	30.07 ±2.33	28.21 ±2.2* ↓	0.24 ± 0.10 ng/mL	0.39 ± 0.17* ng/mL ↑	NP	NP	NP	NP	3.52 ± 0.11	2.33 ± 0.09*# ↓	NP		Myonectin levels↑, BMI, and IR ↓
		(38.89 ± 1.78 yrs)	Control group *n*=46	NP	NP	30.01 ± 2.70	30.07 ± 2.16↑	0.22 ± 0.44 ng/mL	0.23 ± 0.15 ng/mL ↑	NP	NP	NP	NP	3.88 ± 1.3	3.87 ± 1.2# ↓			Myonectin levels and BMI ↑, IR ↓
Safarzade *et al.*, 2017 Obese men	8 weeks	37.0 ± 8.5 years	Group 1: Control *n* = 11	96.3 ± 12.5	96.1 ± 12.9*	32.1 ± 3.0	32.0 ± 3.1*	~50 Pg/mL	~48 Pg/mL	No sig		No sig		NP		202.4± 33.9	191.4 ± 35.4*	Myonectin levels ↓, Body weight, BMI, fasting glucose and fasting insulin ↓
			Group 2: resistance training *n* = 12	95.5 ± 10.3	92.3 ± 9.7*	31.5 ± 2.0	30.5 ± 1.7*	~48 Pg/mL	~55 Pg/mL							196.3± 31.9	167.7± 26.9*	Myonectin levels ↑, Body weight, BMI, fasting glucose and fasting insulin ↓
Bagheri *et al.*, 2018 Overweight women	8 weeks	(29.928± 2.758 yrs)	HIIT group, *n* = 14	74.185± 4.491	72.557 ± 4.581	28.147± 1.126	27.575 ± 1.391	2805.714 ± 411.908 Pg/mL	2559.357 ± 423.813 Pg/mL*	84.285± 5.398	82.857 ± 7.843	4.571 ± 0.415	2.30 ± 0.241*	0.952 ± 0.190	0.862 ± 0.114	NP	NP	Myonectin levels ↓, Body weight, BMI, fasting glucose, fasting insulin, and HOMA-IR ↓
		(30.142 ± 3.526 yrs)	Control group, *n* =14	70.842± 5.926	71.214	27.742± 0.882	27.995 ± 1.061	2952.6422± 436.136 Pg/mL	3055.142 ± 415.082 Pg/mL	80.285± 5.398	85.285 ± 10.283	4.207 ± 0.255	4.471 ± 0.482	0.855 ± 0.155	0.952 ± 0.205			Myonectin levels, Body weight, BMI, fasting glucose, fasting insulin, and HOMA-IR ↑
Zefreei *et al.*, 2020 Obese women**	8 weeks	31.6 ± 7.17	Aerobic exercise group *n* = 10	74.185± 4.491	72.557± 4.581	28.147 ± 1.124	27.575 ± 1.391	0.28 ± 0.11 ng/mL	1.21 ± 0.12 ng/mL*	87.6 ± 33.74	79.4 ± 50.99*	14.4 ± 12.59	11.57 ±17.4*	3.1 ± 21.18	2.1 ± 18.17*	NP	NP	All significant Myonectin levels ↑, Body weight, BMI, fasting glucose, fasting insulin, and HOMA-IR ↓
		32.34 ± 7.03	Control group *n* = 10	70.842± 5.926	71.214 ± 6.124	27.742± 0.882	27.995 ± 1.061	0.0 ± 29.11 ng/mL	0.0 ± 82.12 ng/mL	3.11 ± 18.33	2.14 ± 5.45	15.6 ± 30.52	15.6 ± 20.45	3.5 ± 20.52	NP			No change in myonectin levels, body weight, BMI, fasting glucose, fasting insulin ↓
Safarpour *et al.*, 2021 Postmeuposal overweight women	8 weeks	(55.73 ± 2.66 years	Experimental group, *n* = 12	Overall, 55.73 ± 2.66		Overall 26.72 ± 2.33		###	↑*Sig	###	No significant changes	###	↓sig	###	↓Sig	NP	NP	Significant Myonectin level ↑, insulin and insulin resistance ↓
			Control group, *n* = 12															

Values: Mean ± SD, NP: Not Provided, *Significant at *P* value <0.05, ** Green coffee groups not compared in this table, ### Unable to retrieve.

**Table 5:** PICO framework for studies on exercise and myonectin in healthy individuals

Author, Year	Li *et al.*, 2018	Bahremand *et al.*, 2020	Fereshteh *et al.*, 2019	Kamiński *et al.*, 2019	Abdolreza *et al.*, 2020	Joy *et al.*, 2016
**Study Design**	Cross-sectional studies.	NA	Semi-experimental study	Cross-sectional, single-center	NA	NA
**Population**	T2DM, IGT, Healthy individuals	Healthy young women	Sedentary young men	Young, healthy volunteers	Young men	Healthy, resistance-trained men
**Inclusion criteria:**						
**Age > 18 years**	✓	✓	✓-	✓	NA	✓
**BMI ≥ 25 Kg/m^2^**	-	-		-	-	-
**Comparator and Exposure**						
**Control group**	-	-	-	-	-	-
**Exerci ses group**	✓	✓ (CT and Crossfit)	✓	✓	✓	✓
**Outcomes**						
**Myonectin level**	✓	✓	✓	✓	✓	✓
**Adiponectin**	-	-	-	-	-	-
**FPG**	-	✓	-	✓	-	-
**FPI**	-	✓	-	✓	-	-
Insulin resistance*	-	✓	-	✓	-	-
**Total cholesterol**	-	✓	-	-	-	-

**Table 6:** Type of exercise and exercise dosage parameters in healthy individuals

Study	*N*	Experimental group	Control group	Type of exercise	Duration (Week)	Duration (Time minutes)	Duration (x/Week)	Intensity
Li *et al.*, 2018 (Subgroup analysis)	NA	Healthy young individuals, *n* = 12	NA	A 45-minute bout of aerobic treadmill exercise	0	45 minutes	0	with 60% of maximal oxygen consumption for 45 minutes.
Bahremand *et al.*, 2020 Healthy Young women	30	Healthy young women CrossFit, *n* = 16	Healthy young women CT (*n* = 14)	CrossFit and Concurrent training	8 weeks	20-22.5 10-20 minutes	3x/week	Aerobic and resistance exercise with 5% graded increase from 60 to 80 of max heart rate and 60 to 80 of 1RM Cross fit: high-intensity, low repetition, and high percentage of 1RM, as many rounds as applicable (AMRAP), work out of the day
Fereshteh *et al.*, 2019	14	volunteer non-physical education students *n* = 14	-	Resistance training	4 and 6 weeks	NA	3x/week	6 exercises (bench press, barbell curl, leg press, leg extension, leg curl, wide grip lat pull down), with 3 sets of 8-10-12 repetitions in each exercise and the intensity of 60-70-80%of 1 RM (respectively for 1^st^, 2^nd,^ and 3^rd^ weeks).
Kamiński *et al.*, 2019	29	Young, healthy volunteers Female, *n* = 19 and male, *n* = 10	-	Short-term physical activity (Bruce protocol)	0	~20	0	85% of the predicted maximum heart rate.
Abdolreza *et al.*, 2020	20	Resistance training with blood flow restrictions group (*n* = 10) and Resistance training without blood flow restrictions group (*n* = 10)	-	Resistance training	4 weeks	60MIN	NP	Without restrictions -70%; with restrictions - 30%. 1-3 sets, 15 repetitions
Joy *et al.*, 2016	25	the PLA group (*n* = 11) and TRT group (*n* = 14)	-	Resistance and power training	12 weeks (8 wk-2 wk-2wk)	NP	3 days per week for 8 weeks	standard resistance training phase: 1 muscle hypertrophy, 1 power, 1 strength-oriented workout at 40% RM to 100% RM, and 2-6 sets of Wingates cycle ergometer

**Table 7:** Anthropometrics and biochemical parameters before and after exercise training in healthy individuals

Study description				Body weight (Kg)		BMI (Kg/m^2^)		Myonectin		Fasting Glucose (mg/dL)		Fasting Insulin (µU/mL)		HOMA-IR		TC		Body fat percentage		Overall inference After exercising
Author, year/exercise	Duration	Age	Groups	Before	After	Before	After	Before	After	Before	After	Before	after	Before	after					
Li *et al.*, 2018 nT2DM, IGT, Healthy individuals (60% max 02 consumption 45 min activity, rest)	NP	25 ± 2 years old	nT2DM *n* = 104	NP	NP	24.47 ± 3.17	NP	82.3 ± 47.6 µg/mL	NP	9.76 ± 4.1 mmol/L	NP	11.64 ± 6.46	NP	4.00	NP	5.12 ± 1.08	NP	30.87 ± 9.90	NP	NP
			IGT *n* = 109	NP	NP	24.42 ± 2.98	NP	68.9 ± 46.6µg/mL	NP	6.18 ± 1.7 mmol/L	NP	11.97 ± 8.61	NP	2.54	NP	5.22 ± 1.25	NP	31.57 ± 7.84	NP	
		from 42 to 79 years	Healthy individuals *n* = 128	NP	NP	23 ± 5.31	NP	45.2± 23.5 µg/mL	NP	5.22 ± 0.4 mmol/L	NP	8.49± 3.60	NP	1.87	NP	4.98 ± 0.94	NP	29.49 ± 7.16	NP	
		21 to 23 years	*n* = 12 12 young subjects	NP	NP	21.0 ± 1.2 kg/m^2^	NP	18.2 ± 4.4 µg/L	21.7 ± 5.3 µg/L(NS)	NP	NP	NP	NP	NP	NP	NP	NP	NP	NP	Non-significant myonectin ↑
Bahremand *et al.*, 2020 Healthy Young women	8 weeks	30.8 ± 4.9 years	CrossFit (*n* = 16)	63.3 ± 4.7	61.5 ± 5.3	24.0 ± 1.4	23.3 ± 1.6	3.6 ± 1.1 (pg/mL)	3.6 ± 1.1 (pg/mL) No improvement	78.7 ± 5.7 (mg/dL)	77.4 ± 4.7 (mg/dL)	9.5 ± 4.4 (mIU/mL)	9.0 ± 4.6 (mIU/mL)	1.9 ± 0.9	1.7 ± 0.9	157.7 ± 26.5 (mg/dL)	154.4 ± 24.6 (mg/dL)	~30.5%	~28%	No significant improvement in myonectin and HOMA-IR.
			CT (*n* = 14)	61.1 ± 6.1	59.9 ± 6.0	22.7 ± 1.9	22.2 ± 1.8	3.7 ± 0.9 (pg/mL)	3.7 ± 0.8 (pg/mL)	81.0 ± 6.4 (mg/dL)	81.3 ± 7.4 (mg/dL)	9.7 ± 2.4 (mIU/mL)	9.6 ± 3.4 (mIU/mL)	1.9 ± 0.5	1.9 ± 0.7	147.8 ± 23.9 (mg/dL)	138.4 ± 12.0 (mg/dL)	~30%	~27%	No significant improvement in myonectin and HOMA-IR.
Fereshteh *et al.*, 2019 Sedentary young men	4 and 6 weeks	26. 50± 0. 94	Volunteer non-physical education students *n* = 14	NP	NP	23.70 ± 2.34	NP	NP	After 4 weeks: no significant difference. After 6 weeks: increased significantly.	NP	NP	NP	NP	NP	NP	NP	NP	NP	NP	After 4 weeks: no significant difference. After 6 weeks: increased significantly.
Kamińs *et al.*, 2019 Young, healthy volunteers	Short-term Physical activity 11.8 MET MAX ON treadmill	22 (20-23) years old Female:19, male 10	No group	59(54-78)	NP 0.03* Changes sig associated with myonectin	21.3(19.4-24)	NP 0.01* Changes sig associated with myonectin	0.67 (0.14–2.9) ng/ml	1.08 (0.15–2.44) ng/ml	5.1(4.8-5.3)	NP	16.1(12.5-19.1)	0.04* Changes sig associated with myonectin	3.7(2.8-4.3)	0.02* Changes sig associated with myonectin	4.4(3.9-5.2)	NP	NP	NP	Not significant Myonectin levels ↑
Abdolreza *et al.*, 2020 Young men	4 weeks	NP	Resistance training with blood flow restrictions group (*n* = 10) hardship 30% (1hr) 4 weeks	73.4 ±5.3	73.3± 2.9	23.1 ± 37.35	23.0 ± 36.87	512.0± 37.46 ng/L	528 ± 26.74 ng/L	NP	NP	NP	NP	NP	NP	NP	NP	NP		Myonectin levels: no significant increase
		NP	Resistance training without blood flow restrictions group (*n* = 10) hardship 70%((1hr) 3 sessions for week 4 weeks	77.6 ± 2.8	76.6± 7.3	24.2 ± 4.11	24.1 ± 36.97	512.0± 74.43	522.3 ± 90.54											
Joy *et al.*, 2016 Healthy, Resistance- trained men	12 weeks	28 ± 5 years old	*n* = 25 the PLA group (*n* = 11)	83.2 ± 12.1	NP	NP	NP	0.26 ± 0.14 ng/mL	0.36 ± 0.46 ng/mL	NP	NP	NP	NP	NP	NP	NP	NP	NP		Myonectin levels ↑ Nonsignificant in both groups.
								0.81 ± 1.52 ng/mL	0.99 ± 2.05 ng/mL											
			TRT group (*n* = 14)																	

Values: Mean ± SD, NP: Not Provided, *Significant at *P* value <0.05, HOMA-IR, Homeostatic Model Assessment for Insulin Resistance.

### Summary of myonectin level changes and exercise associated with insulin resistance for exercise interventions longer than 8 weeks

Two studies investigated the association between myonectin level changes and insulin resistance following exercise interventions in different populations. Lim *et al*. [24] studied healthy younger women (22.5 years old) and older overweight women (60.3 years old). They found that exercise significantly decreased myonectin levels in both groups. Choi *et al*. [25] investigated women with obesity (30-60 years old). They found that exercise significantly increased myonectin levels in this population. Overall, the findings of these two studies appear contradictory. The observed differences may be due to variations in the baseline characteristics of the study populations, such as age and weight status. More research is needed to elucidate the complex relationship between myonectin, exercise, and insulin resistance. Exercise decreased myonectin levels in healthy, younger, and older overweight women, while it increased myonectin levels in obese women, following combined resistance and aerobic exercise of 12 weeks.

### Summary of myonectin level changes and their association with insulin resistance in 8-week exercise interventions

Significant increases in myonectin levels were observed after exercise in two studies [30-31], which involved exercise intensities of 60% to 70% of maximum heart rate. These protocols were comparable to swimming exercises and targeted postmenopausal overweight and obese women. This increase in myonectin levels was also associated with significant changes in fasting insulin and insulin resistance. In contrast, concurrent training or resistance training [27,28] relatively increased myonectin levels in obese and overweight older women and obese men, though not statistically significant. This non-significant increase could not translate into significant changes in fasting glucose, insulin, and insulin resistance, except in the group that underwent resistance endurance, which showed a significant reduction in fasting glucose. Weight and BMI also reduced significantly after 8 weeks of exercise training. Resistance training was associated with a significant decrease in total cholesterol and body fat percentage.

Interval running with the maximum speed, using the high-intensity interval training (HIIT) principle and characterized by near-exhaustive effort [29], decreased the myonectin levels in overweight, inactive women. Fasting insulin levels showed significant beneficial changes in the HIIT group, with a notable reduction. However, no significant changes in insulin resistance were observed. Hence, exhaustive exercise prescription warrants more research.

- Myonectin levels generally increased after exercise interventions across various populations, including older women, obese men, overweight women, and postmenopausal women, in 5 out of 6 studies included.
- In most studies, exercise was consistently associated with improvements in insulin resistance (decreased fasting glucose, fasting insulin, and HOMA-IR) alongside myonectin level increases.
- Different exercise modalities (endurance-resistance, aerobic, HIIT, and resistance training) showed similar trends of myonectin level increases and improved metabolic parameters.

These findings suggest that exercise interventions increase myonectin levels and may contribute to improved insulin sensitivity and metabolic health in diverse populations. Control groups often showed either stable or slightly decreased myonectin levels, emphasizing the role of exercise in modulating myonectin production and metabolic health. Myonectin levels and insulin sensitivity respond differently to exercise duration among specific populations. Shorter-duration, high-intensity exercises (like HIIT) can decrease myonectin while improving insulin sensitivity, especially in overweight, inactive women. Longer-duration, moderate-intensity exercises, especially combined resistance and aerobic training, tend to increase myonectin levels and further enhance insulin sensitivity, particularly in older women, obese men, and overweight women. More research is needed to fully understand the complex interplay between exercise duration, myonectin, and metabolic health across diverse populations.

### Exercise choices related to myonectin levels and metabolic health

Based on the number of studies and the consistency of findings, aerobic exercise emerged as the most supported exercise for increasing myonectin levels. In three of the eight reviewed studies, aerobic exercise was used and consistently demonstrated positive effects on myonectin levels across diverse populations and study designs [24,26,30].

Other exercise modalities, such as HIIT [29,31] and combined resistance and aerobic exercise [25], also showed potential in influencing myonectin levels. However, these modalities were examined in fewer studies compared to aerobic exercise. Resistance training, while beneficial for other aspects of metabolic health, showed mixed findings regarding its effect on myonectin levels [27,28]. As seen in HIIT protocols, short bursts of maximum-speed interval training may have specific applications in overweight and obese populations but should be approached cautiously. Based on the available evidence, aerobic exercise appears to be the most effective and consistently supported modality for increasing myonectin levels.

Based on the data from studies involving healthy participants, no specific exercise interventions, including CrossFit, circuit training (CT), short-term physical activity, or resistance training with or without blood flow restrictions, consistently demonstrated significant increases in myonectin levels. Some studies reported no significant changes, while others noted some changes associated with exercise but not consistently significant. Therefore, the available evidence does not clearly identify a specific exercise type superior for increasing myonectin levels in healthy participants. More targeted research may be needed to establish effective exercise interventions.

**Figure 2:**
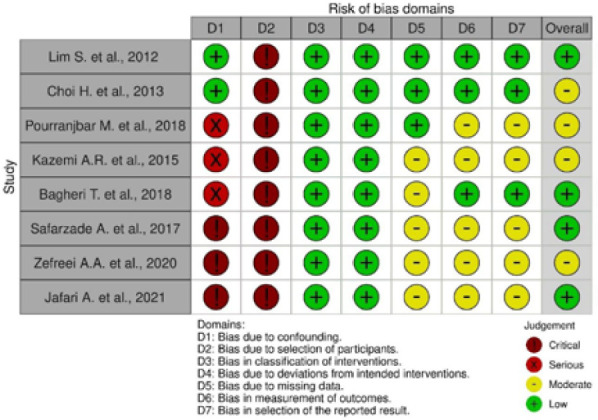
Risk of bias summary (studies including obese and overweight participants)

The ROBINS-I tool was used to systematically evaluate bias in the non-randomized studies included in our study, covering confounding, participant selection, intervention classification, deviations from intended interventions, missing data, outcome measurement, and selective reporting. Most studies showed serious to critical risk of bias in domains related to confounding and selection of participants. Most studies had a low risk of bias in domains such as intervention and deviations from intended interventions, suggesting robust methods in those areas. The overall risk of bias ranged from moderate to serious across the studies (Figure 2).

**Figure 3:**
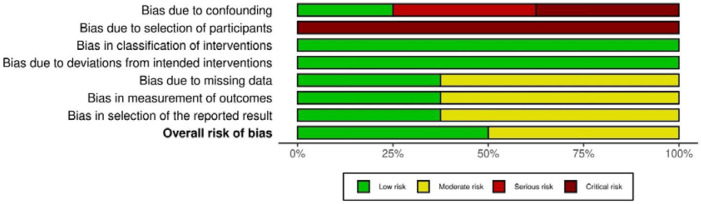
Risk of bias graph (studies including obese and overweight participants)

The risk of bias graph (Figure 3) highlights areas of concern and strength across various domains for multiple studies.

### High-risk areas

Confounding and participant selection were the most problematic areas, with many studies exhibiting serious to critical risks of bias. Figure 3 suggests that future research should focus on improving control of confounding factors and the selection process of participants in myonectin studies.

### Low-risk areas

Intervention classification, adherence to intended interventions, and outcome measurement generally showed low risks of bias, indicating strong performance in these domains, ensuring robust exercise intervention in selected populations.

## DISCUSSION

**Figure 4:**
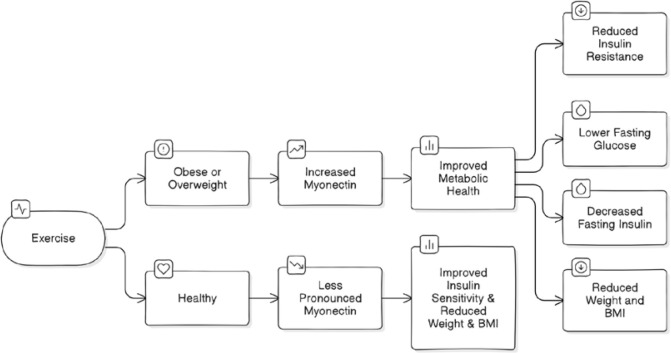
Exercise, Myonectin & Metabolic Health Flowchart

**Figure 5:**
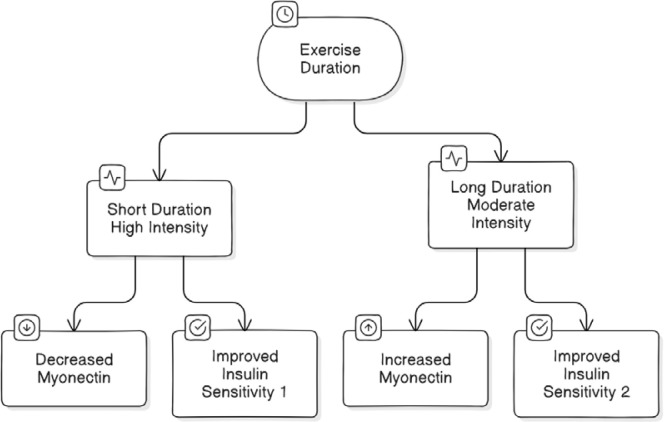
Exercise Duration, Myonectin and Insulin Sensitivity Flowchart

The elevation in myonectin may contribute to immediate improvements in insulin sensitivity and glucose metabolism by facilitating nutrient uptake and utilization in skeletal muscle [38,39]. Figures 4 and 5 provide a comprehensive overview of the overall effects of exercise on myonectin and metabolic health, as well as the duration-dependent outcomes of exercise interventions.

### A conundrum with adiposity

The myonectin response to exercise appears modulated by adiposity status, with obese individuals exhibiting blunted or delayed responses compared to their lean counterparts. This impaired myonectin regulation in obesity may reflect underlying disturbances in skeletal muscle metabolism and adipose tissue function [40]. However, exercise interventions tailored to overweight and obese individuals have been shown to effectively enhance myonectin expression and improve insulin sensitivity, highlighting the therapeutic potential of physical activity in these groups [41].The key difference seems to be that exercise has a more pronounced effect on increasing myonectin in individuals who are overweight or obese, likely due to the role of myonectin in regulating lipid metabolism and insulin sensitivity in these populations. In healthy, lean individuals, the exercise-induced changes in myonectin are less clear. In summary, the search results indicate that exercise, both aerobic and resistance, can significantly increase myonectin and improve insulin resistance in overweight and obese individuals, but the effects are less consistent in healthy, non-obese adults. The reasons why myonectin levels respond differently to exercise in healthy adults compared to overweight/obese adults are not fully understood. Genetic expression, diet, energy usage during exercise, and other complex myokines or adipokines interplay were factors that needed to be considered. The studies suggest that the exercise-induced increase in myonectin and its beneficial effects on lipid metabolism and insulin sensitivity are more pronounced in overweight/obese populations than in healthy, lean individuals [42,43].

Mechanistically, myonectin appears to promote fatty acid uptake in adipose tissue and the liver, thereby regulating systemic lipid homeostasis. Myonectin can increase the expression of genes involved in lipid uptake and transport, such as CD36, FATP1, Fabp1, and Fabp4 [43,44].

In obese and insulin-resistant states, the reduced levels of myonectin may contribute to increased central obesity and lipid accumulation. Restoring myonectin levels through exercise may help improve lipid metabolism and insulin sensitivity in these populations [43,13]. While the scientific reasons for the difference are not fully understood, the key takeaway is that exercise is beneficial for young adults in improving insulin sensitivity and overall health. Exercise-induced increases in myonectin levels may contribute to improved insulin sensitivity and glucose uptake in skeletal muscle, reducing insulin resistance and the risk of developing T2DM [45]. Additionally, exercise has been shown to enhance mitochondrial density and function, further improving metabolic health and insulin sensitivity among overweight and obese individuals [46].

### Aging effect and myonectin

The mean age of the participants was 21 to 30.8 years for healthy individuals. Exercise inconsistently affected myonectin levels, with minimal changes and little impact on key metabolic parameters. This aspect warrants more research. 12-week interventions showed more consistent improvements in myonectin levels associated with metabolic benefits compared to 8-week interventions among individuals aged between 22.5 and 60.3 years.

### Exercise, myonectin, and metabolic health indicators

Studies also reported improvements in fasting plasma glucose, fasting plasma insulin, and total cholesterol levels following exercise interventions and their association with myonectin levels. However, there was limited data on other parameters, such as hemoglobin A1c levels, which hindered the evaluation of myonectin as a potential biomarker for prediabetes or diabetes screening [14,47], as well as exercise prescription.

### Diabetes and myonectin conundrum

In patients with diabetes, lower myonectin levels are associated with worse metabolic parameters like higher BMI, cholesterol, triglycerides, insulin resistance, and inflammation. Similarly, obese non-diabetic individuals have significantly lower myonectin levels compared to lean controls [48]. However, myonectin levels exhibit a progressive increase from normal glucose tolerance to prediabetes to T2DM, with the highest levels observed in overt diabetes [16].

Based on the available evidence, our findings suggest that myonectin may serve as a potential biomarker for diabetes risk and insulin resistance, particularly in overweight and obese individuals. Given its role in metabolic regulation, aerobic exercise, combined resistance, aerobic exercise, and HIIT appear to be beneficial interventions. This type of exercise can improve insulin sensitivity, manage metabolic stress, and potentially influence myonectin levels favorably, especially in populations at risk for diabetes or with existing insulin resistance.

To replicate our findings and mitigate time delays and gaps in current literature, we conducted an extended search using similar keywords and search strategies in the PubMed database for studies published between 2022 and 2024. The search strategy was: [(("Exercise"[MeSH Terms] OR "Physical Activity" OR "Resistance Training" OR "Aerobic Exercise" OR "High-Intensity Interval Training" OR "HIIT") AND ("Myonectin" OR "CTRP15" OR "C1q/TNF-related protein 15" OR "Myokines" OR "Adipokines") AND ("Insulin resistance"[MeSH Terms] OR "Glucose Metabolism" OR "Glycemic Control" OR "Type 2 Diabetes" OR "Metabolic Syndrome") AND ("Overweight"[MeSH Terms] OR "Obesity"[MeSH Terms] OR "Healthy" OR "Lean" OR "Elderly" OR "Normal Weight")) NOT ("Diabetes"))]. This search resulted in 16 articles, of which only 2 met our inclusion criteria for investigating exercise, myonectin, and insulin resistance in healthy, obese, and overweight individuals.

A study by Bahremand [33], available as a preprint online in 2020 and published in 2023, has already been included in our thematic synthesis. A recent study replicated our findings [49]. The study concluded that in sedentary individuals with metabolic syndrome, myonectin levels are typically reduced compared to non-metabolic individuals. Exercise, particularly HIIT, generally increased myonectin levels in both groups. However, the increase was often more significant in those with metabolic syndrome, improving lipid metabolism and metabolic health, and also negatively correlated with the android/gynoid fat mass ratio. No correlation was found in lipid profile, free fatty acids (FFA), intramuscular lipid content, or HOMA-IR [49]. However, these findings are limited to a single database. Expanding searches to additional databases may provide additional cues.

### Limitations

Despite providing insights, this review has several limitations. Firstly, significant heterogeneity among the included studies, particularly in methodological quality, may impact the reliability of the findings. The evidence of certain effects was limited due to small sample sizes, lack of blinding, and potential bias presented in the included studies. Hence, knowledge of evidence synthesis needs to be interpreted with caution. Additionally, there was a lack of uniformity in exercise interventions, with variations in duration, intensity, and type of exercise across studies, making it challenging to draw definitive conclusions. Furthermore, the absence of studies focusing specifically on pre-diabetic or diabetic populations limits the generalizability of the findings to these groups. Moreover, the review identified a scarcity of longitudinal clinical studies, highlighting the need for more robust research in this area. Lastly, the limited availability of data on certain parameters, such as hemoglobin A1c levels, hindered a comprehensive evaluation of the relationship between myonectin, exercise, and glycemic control. These limitations underscore the necessity for future studies to address methodological inconsistencies, expand to other databases and grey literature, explore diverse populations, and provide comprehensive data to enhance the understanding of the impact of exercise on myonectin and insulin resistance among overweight and obese individuals. A cautious interpretation of current findings is warranted due to the non-randomized quasi-experiments and cross-sectional design of studies included in the current review. Overall, while the studies show strengths in several areas of bias assessment, significant attention is needed to address confounding and participant selection biases to improve the reliability and validity of non-randomized studies. This can be addressed by randomized controlled studies.

### Future recommendations

Future research should explore several key areas to deepen our understanding of the impact of exercise on myonectin and insulin resistance among overweight and obese individuals. Firstly, long-term studies investigating the effects of various exercise modalities, intensities, and durations on myonectin regulation and insulin resistance markers in diverse populations are essential. Longitudinal studies with larger sample sizes and well-controlled clinical designs could provide more robust evidence on the role of exercise in preventing the transition from overweight/obesity to prediabetes.

Few selected studies included HbA1c measurements despite exercise interventions lasting up to 12 weeks. As a result, this review could not fully assess myonectin as a biomarker for prediabetes risk or exercise response. More studies are suggested to address this biomarker linkage. Given its potential role in insulin resistance, investigating the use of myonectin measurements in clinical practice to monitor overweight/obese patients’ symptoms and guide exercise prescriptions for improved metabolic health is warranted.

Studies comparing different exercise modalities (e.g., constant-moderate vs. HIIT) and their impact on myonectin levels and insulin resistance in humans with prediabetes and T2DM are needed. The hepato-protective effects on beta-cells of various myokines exerted by exercise interventions and their influence on diabetic transition and complications must also be explored.

## CONCLUSION

Studies show mixed results on how exercise affects myonectin levels and insulin resistance. In overweight and obese individuals, aerobic exercise, combined resistance, aerobic exercise, and HIIT consistently elevated myonectin levels alongside improved BMI, fasting glucose, fasting insulin, and HOMA-IR, indicating enhanced metabolic health. Conversely, evidence in healthy individuals regarding myonectin response to exercise is inconclusive, with some studies showing no significant changes, highlighting potential variability based on baseline metabolic status. In conclusion, while aerobic exercise appears beneficial for increasing myonectin levels and improving metabolic health, combined resistance, aerobic exercise, and HIIT also show promise. These exercise modalities can play a crucial role in managing insulin resistance and may prevent transitioning into pre-diabetes, particularly in overweight and obese populations. Further research is needed to understand how exercise impacts myonectin levels in healthy individuals and to optimize exercise prescriptions for metabolic health.

## Personal thanks

The authors would like to thank the Research Office and GMU Library for their support in providing access to resources and assistance in retrieving information in the Persian language.
