# Supplementary material for: Exercise, myonectin response, and insulin resistance among overweight, obese and healthy individuals: a systematic review and narrative synthesis
Source: J Med Life. 2025 Feb;18(2):73–89. doi: 10.25122/jml-2024-0277 (PMC11932511; doi:10.25122/jml-2024-0277)
Supplement: Supplementary file 1 [file JMedLife-18-073-s001.pdf]

SUPPLEMENTARY SEARCH TERMS AND DATABASE

PubMed:

(("Exercise"[MeSH Terms] OR "Physical Activity" OR "Resistance Training" OR "Aerobic Exercise" OR "High-Intensity Interval Training" OR "HIIT") AND ("Myonectin" OR "CTRP15" OR "C1q/TNF-related protein 15" OR "Myokines" OR "Adipokines") AND ("Insulin Resistance"[MeSH Terms] OR "Glucose Metabolism" OR "Glycemic Control" OR "Type 2 Diabetes" OR "Metabolic Syndrome") AND ("Overweight"[MeSH Terms] OR "Obesity"[MeSH Terms] OR "Healthy" OR "Lean" OR "Elderly" OR "Normal Weight"))
Filters: (2000 to 2022 - 214) (2022 to 2024- 22)

Cochrane Library:

("exercise" OR "physical activity" OR "resistance training" OR "aerobic exercise" OR "high intensity interval training" OR "HIIT") AND ("myonectin" OR "CTRP15" OR "C1q TNF related protein 15" OR "myokine" OR "myokines" OR "adipokine" OR "adipokines") AND ("insulin resistance" OR "glucose metabolism" OR "glycemic control" OR "type 2 diabetes" OR "metabolic syndrome") AND ("overweight" OR "obesity" OR "healthy" OR "lean" OR "elderly" OR "normal weight")
(1995-2022 - 160) (2022 to 2024 - 33)

CINHAL/EBSCO:

("exercise" OR "physical activity" OR "resistance training" OR "aerobic exercise" OR "high intensity interval training" OR "HIIT") AND ("myonectin" OR "CTRP15" OR "C1q TNF related protein 15" OR "myokine" OR "myokines" OR "adipokine" OR "adipokines") AND ("insulin resistance" OR "glucose metabolism" OR "glycemic control" OR "type 2 diabetes" OR "metabolic syndrome") AND ("overweight" OR "obesity" OR "healthy" OR "lean" OR "elderly" OR "normal weight")
(2000-2022)21

Science direct via SCOPUS:

("exercise")AND ("myonectin" OR "C1q TNF related protein 15")AND ("insulin resistance" OR "insulin sensitivity")AND ("overweight" OR "obese" OR "healthy")
(1995-2022 - 44) (2022-2024 - 20)

Google scholar:

"exercise" OR "physical activity" OR "resistance training" OR "aerobic exercise" OR "HIIT" AND "myonectin" OR "CTRP15" OR "myokine" OR "adipokine" AND "insulin resistance" OR "type 2 diabetes" OR "metabolic syndrome" AND "overweight" OR "obese" OR "healthy individuals" OR "elderly"
(2012-2022- 8610) (2022-2024- 3910)

TRIP database:

(exercise OR "physical activity" OR "resistance training") AND (myonectin OR ctrp15 OR "c1q tnf related protein 15") AND ("insulin resistance" OR "insulin sensitivity" OR ") AND (overweight OR obese OR healthy)
(1995-2022 -15) (2022 to 2024- 1)

© 2025 by the authors. This article is an open access article distributed under the terms and conditions of the Creative Commons Attribution (CC BY 4.0) license.
